# Incidence of Antenatal Trichomoniasis and Evaluation of Its Role as a Cause of Preterm Birth in Pregnant Women Referring to Minia University Hospital, Egypt

**Published:** 2018

**Authors:** Amany Mohamed KAMAL, Azza Kamal AHMED, Nawras Mohamed El-Saghier MOWAFY, Hossam Eldin SHAWKI, Ahmed Samir SANAD, Eptesam Esmail HASSAN

**Affiliations:** 1. Dept. of Parasitology, Faculty of Medicine, Minia University, Minia, Egypt; 2. Dep. of Parasitology, Faculty of Medicine, Umm Al-Qura University, Mecca, Saudi Arabia; 3. Dept. of Obstetrics and Gynecology, Faculty of Medicine, Minia University, Minia, Egypt; 4. Dept. of Public Health and Preventive Medicine, Faculty of Medicine, Minia University, Minia, Egypt

**Keywords:** *Trichomonas vaginalis*, Diagnosis, Preterm delivery, Neonatal outcome, Risk factors, Egypt

## Abstract

**Background:**

We aimed to determine the incidence of trichomoniasis and its risk factors in Egyptian pregnant women attending the Minia Maternity and Pediatric University Hospital, Minia, Egypt and evaluate its association with preterm birth.

**Methods:**

The study was carried out from Aug 2014 to Jun 2015 through 2 phases, the first phase was case-control study, and the second phase was follow-up with intervention. Overall, 300 pregnant women with gestational age of 20–36 weeks with no medical risk factors of preterm labour birth were enrolled. Vaginal swabs were examined by the wet mount microscopy and culture while urine samples were examined by urine analysis. Demographic information was collected. Pregnant women were divided into two groups, study group (with trichomoniasis) and control group (without trichomoniasis). Positive cases were subjected to metronidazole treatment.

**Results:**

Thirty-five cases were positive for *T. vaginalis* infection. Maximum cases were detected by culture (11.7%) followed by wet mount microscopy (9.7%) whereas least number of cases (7.3%) was detected by urine examination. Nineteen (54.28%) cases had preterm delivery. Post-delivery adverse outcomes were observed in 29 cases (82.8%). The high rate of infection was observed in age group of 20–30 years (*P*<0.05). In addition, there was a significant *T. vaginalis* infection in pregnant women living in rural area, of low socioeconomic and primary educational levels (*P*<0.05).

**Conclusion:**

Pregnant women lived in rural area with a low socioeconomic and primary educational levels should be screened for trichomoniasis to reduce the incidence of preterm delivery and low birth weight.

## Introduction

Human trichomoniasis is a common vaginal infection caused by the flagellate protozoan *Trichomonas vaginalis* with an annual incidence of 180 million cases (1).

Preterm birth and low birth weight are considered one of the most important causes of neonatal mortality and morbidity worldwide. Low birth weight is responsible for more than 60% of mortality among infants without congenital anomalies (2). *T. vaginalis* infection has been considered as an important cause of preterm birth and low birth weight (3).

Although trichomoniasis is usually manifested by vaginal discharge, dysuria, itching, vulvar irritation and abdominal pain up to 50% of infected women are asymptomatic (4). Laboratory tests play the major role in diagnosis of trichomoniasis. Wet mount and culture techniques are the most widely used methods. Although wet mount is simple and cheap, the sensitivity of culture is higher ranging from 44% to 95% (5).

The aims of this study were to determine the incidence of *T. vaginalis* infection in Egyptian pregnant women attending the antenatal care clinic of Minia Maternity and Pediatric University Hospital, Minia, Egypt by using different diagnostic techniques and to evaluate the association between the infection and preterm birth and its risk factors.

## Materials and Methods

### Study design and setting

This study was carried out between Aug 2014 and Jun 2015 at the antenatal care clinic of Minia Maternity and Pediatric University Hospital, Minia, Egypt by two phases:

**Phase I:** Case-control study including selection of the participants as follows:

Inclusion criteria: antenatal women of reproductive age with or without vaginal discharge.

Exclusion criteria: Women with systemic diseases (e.g., diabetes mellitus, hypertension, renal, or heart disease), uterine anomalies, incompetent cervix, excess uterine contraction, oversized uterus (polyhydramnios, multiple pregnancies), abruption placenta, cervical bacterial infection, previous history of preterm labor, premature rupture of membrane, prior use of tocolytic and corticosteroid agents during the current pregnancy, or use of antibiotics in the preceding two weeks, premature rupture of membrane were excluded from this study.

Gestational age was calculated according to the last menstrual period date and confirmed ultrasonography (6).

A total of 300 pregnant women within the age range of 18–40 yr (mean age 28.3±6.1yr) with gestational age of 20–36 wk, with or without complaint of vaginal discharge were enrolled in the present study. According to *Trichomonas vaginalis* infection result, pregnant women were divided into two groups, study group (Pregnant women with trichomoniasis) and control group (Pregnant women without trichomoniasis).

### Data collection

Socio-demographic information was obtained from participants by use of structured questionnaire. The questionnaires were applied at face-to-face interviews by the researchers. The questions were answered orally by the participants and recorded by the researchers. The participant's name was written on the questionnaire so that the risk of interviewing the same patient was eliminated. Delivery data, neonatal outcome, and medical records were obtained from hospital discharge records to collect information about labor complications and neonatal outcomes.

### Laboratory procedure

Vaginal swabs and urine sample were collected from each pregnant woman at the time of enrollment in the study.

Vaginal secretion was collected from the posterior fornix on two sterile cotton-tipped swabs. The first swab was placed in 2 ml of sterile PBS (pH 7.3), agitated and examined microscopically immediately for motile *T. vaginalis* parasite. Briefly, one drop of saline mixture was aspirated by a Pasteur pipette, put on a microscopic slide, covered with a cover slip and was examined with ×10 and ×40 objectives of a light microscope searching for motile flagellates. Each sample was examined for 3 min. The trophozoites of *T. vaginalis* were identified by their size (10–20 μm), oval shape and characteristic twitching motility (7).

The second swab was inoculated immediately into the culture tube containing 10 ml Diamond modified broth prepared in our parasitology laboratory according to the manufacturer’s instruction (8). The PH of medium was adjusted to 6.0 with 1 N NaOH and prepared medium was sterilized by autoclaving at 121 °C for 15 min. After cooling, sterile inactivated horse serum and the antibiotics (1000 I.U./ml of Penicillin G, 500 μg/ml of Streptomycin sulfate, 250 units/ml of Mycostatin) were added to suppress bacterial and fungal growth. Before vaginal swabs were placed into the medium, culture tubes were warmed to 37 °C or 15 min. All samples were observed microscopically for the presence of *T. vaginalis* trophozoites daily for 7 d.

Aseptically, 10 ml of well-mixed urine was transferred to labeled test tube and was centrifuged at 3000 × gr for 10 min. One drop from the sediment was placed on a microscopic slide and covered with a coverslip. The whole coverslip area was examined with ×10 and ×40 objectives of a light microscope searching for motile *Trichomonas* flagellates (7).

### Phase II: follow-up with intervention

All positive cases were treated. Treatment regimens were prescribed according to US Center for Disease Control’s Sexually Transmitted Diseases treatment guidelines 2010, with metronidazole administered orally 500mg twice a day for 7 d (9). Furthermore, positive cases were followed-up to assess the outcome of their pregnancy.

### Ethical approval

The study protocol was approved by Ethics Committee of the Department of Parasitology and the Department of Obstetrics and Gynecology, Faculty of Medicine, Minia University at their monthly meeting in May 2014. A written informed consent was obtained from each participant.

### Statistical analysis of data

The collected data tabulated and analyzed using SPSS ver. 19 software (Inc., Chicago, ILL Company). Data presented as number and percentages. The Chi-square (test of proportion) and Fisher's exact test used as a test of significance. Receiver operating characteristic curve (ROC) used to determine sensitivity and specificity of direct wet mount and urine test compared with Diamond modified broth culture for diagnosis of vaginal trichomoniasis. Two-sided *P*<0.05 was considered significant. Logistic regression analysis and odds ratio were calculated to see the effect of different independent variables on the target (dependent variable).

## Results

Out of 300 pregnant women enrolled in our study, 35 cases were positive for *T. vaginalis* infection. Maximum cases were detected by culture (11.7%) followed by wet mount microscopy (9.7%) whereas least number of cases (7.3%) was detected by urine examination (Table 1).

**Table 1:** Accuracy of vaginal wet mount and urine tests compared to culture as a reference test for diagnosis of *T. vaginalis* in pregnant women referring to Minia University Hospital, Egypt

		***Culture (Diamond modified broth)***			***Accuracy measure***			
		**Positive (n=35)**	**Negative (n=265)**	**Total (n=300)**	**Variable**	**%**	**AUC (95%CI)**	***P*-value**
Wet mount	Positive	29	-	29	Sensitivity	82.9%	0.91±0.03 (0.88-0.98)	0.001
	Negative	6	265	271	Specificity	100%		
	Total	35	265	300	PPV	100%		
					NPV	97.8%		
Urine	Positive	22	-	22	Sensitivity	62.9%	0.81±0.05 (0.71–0.91)	0.001
	Negative	13	265	278	Specificity	100%		
	Total	35	265	300	PPV	100%		
					NPV	95.3%		

PPV: Positive predictive value

NPV: Negative predictive value

AUC: Area Under Curve

CI: Confidence interval, *P-*value < 0.05

**Fig. 1:**
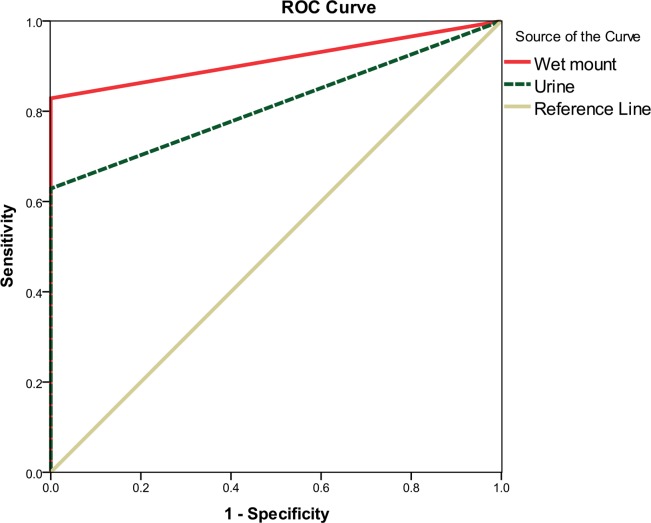
Receiver operating characteristic curve (ROC) analysis of the methods used in diagnosis of vaginal trichomoniasis in pregnant women referring to Minia University Hospital, Egypt

The evaluation of validity and Area under Curve (AUC) analysis of sensitivity and specificity of wet mount microscopy and urine against the gold standard culture were shown in Table 1 and Fig. 1.

Among the positive cases, 19 (54.28) cases had preterm delivery, 14 (40) delivered at term and only 2 (5.7) cases had post-term delivery (Table 2).

**Table 2:** Distribution of *T. vaginalis* infection among the pregnant women with respect to terms of delivery in referring women to Minia University Hospital, Egypt

***Terms of Delivery***	***Trichomonas vaginalis infection***				***P-value***
	**Study group (positive cases)**		**Control group (negative cases)**		
	**No.**	**(%)**	**No.**	**(%)**	
Preterm birth	19	(54.28)	36	(13.58)	0.001
Full term birth	14	(40)	216	(81.5)	0.001
Post term birth	2	(5.7)	13	(4.9)	0.83
Total	35	(100)	265	(100)	

(*P*-value < 0.05 is significant)

Post-delivery adverse outcomes were observed in 29 cases (82.8%). Low birth weight was the most common form of pregnancy wastage (26 cases; 74%) (Table 3).

**Table 3:** Clinical obstetric outcome of pregnancy in pregnant women with *T. vaginalis* infection referring to Minia University Hospital, Egypt

***Clinical obstetric outcome***	***Infants of infected mothers (study group)***		***Infants of non-infected mothers (control group)***		***P-value***
	**No**	**(%)**	**No**	**(%)**	
Low birth weight^a^	19	(54.2)	27	(10.1)	0.001
Preterm low birth weight^a^	7	(20)	14	(5.2)	0.003
Still birth (IUFD)	1	(2.85)	9	(3.39)	0.86
Small for gestational age (SGA)	2	(5.7)	6	(2.26)	0.23
Congenital anomalies	0	(0.00)	2	(0.75)	0.60
Neonatal death	0	(0.00)	3	(1.1)	0.52
Normal baby	6	(17.1)	204	(76.9)	0.001
Total	35	(100)	265	(100)	

a Low birth weight (Low birth weight + Preterm low birth weight) // (*P*-value < 0.05 is significant)

Assessment of the sociodemographic risk factors among the *T. vaginalis* positive cases was performed (Table 4). The high rate of infection was observed in age group of 20–30 yr that was statistically significant compared to other age groups (*P*<0.05). In addition, there was a significant *T. vaginalis* infection in pregnant women living in rural area, of low socioeconomic status, homemaker and with primary educational level.

**Table 4:** Logistic regression analysis of sociodemographic risk factors for vaginal trichomoniasis positivity in pregnant women

*Demographic characterization*	*Total number N (%)*	*No. of T. vaginalis negative cases (%)*	*No. of T. vaginalis positive cases (%)*	*OR (95% CI)*	*P-value*
Age(yr)					
>20	44(14.7)	2 (5.7)	42 (15.8)	3.2 (1.5–8.6)	0.002*
20–30	100(33.3)	22(62.9)	78 (29.4)		
31–40	156(52)	11(31.4)	145(54.7%)		
Residence					
Rural	126(42)	23(65.7)	103(38.9)	3.01 (1.4–6.1)	0.003*
Urban	174(58)	12(34.3)	162(61.1)		
Education level					
Illiterate	8 (2.7)	3(8.6)	5 (1.9)	5.4 (1.2–24.1)	0.02*
Primary	71(23.7)	14(40)	57(21.5)		
Secondary	128(42.7)	10(28.6)	118(44.5)		
University	93 (31)	8(22.9)	85(32.1)		
Occupation					
Not working	147(49)	22(62.9)	125(47.2)	2.3 (1.1–4.8)	0.02*
Working	153(51)	13(37.1)	140(52.8)		
Socio-economic level					
Low	108(36)	21(60)	87 (32.8)	3.6 (1.7–7.4)	0.001*
Middle	159(53)	11(31.4)	148(55.8)		
High	33(11)	3(8.6)	30(11.3)		
Vaginal discharge					
Yes	111(37)	19(54.3)	92 (34.7)	2.6 (1.3–5.4)	0.007*
No	189(63)	16(45.7)	173(65.3)		

OR: odds ratio, CI: confidence interval, *P-*value < 0.05

## Discussion

The present study showed that the incidence of trichomoniasis among pregnant women was 11.7% (35/300). There is no report about infection in pregnant women in Egypt but several studies were done on non-pregnant women indicated almost similar incidence (10–12). Vaginal wet mount microscopy had a better detection than urine microscopy (9.6% versus 7.3%) indicating that in women, routine laboratory could be better achieved by vaginal wet mount microscopy rather than urine analysis. This result was in agreement with other previous studies (13, 14).

The Diamond modified broth culture has been considered the gold standard test according to many studies (15–17). Diamond’s medium results were reported in maximum parasite growth in vitro (18). In the present study, the Diamond modified broth culture was used as the reference test, to which other tests were compared. When wet mount microscopy compared with culture, its sensitivity was found to be 82.9% and the calculated AUC was (0.91±0.03) indicated that wet mount accuracy comes directly next to that of Diamond's culture and has high ability to discriminate between those individuals with or without the disease. Wet mount is simple, inexpensive specific provides immediate results and needing only a microscope and a trained personnel. However, it has some disadvantages as more than 10^3^/ml of living parasites are required for detection (19), sluggish motility of *T. vaginalis* can be easily missed. In addition, vaginal douches before physical examination can decrease the chance of detection of motile *T. vaginalis* (20). Therefore, the combination of wet mount and culture is still the standard approach for more effective diagnosis, especially in asymptomatic patients.

Although Mabey et al. (21) emphasized the importance of maintaining the culture medium for 7–10 d after inoculation. In our study, parasite positive culture was observed in the first 12 h following inoculation and the largest number was identified within 48 h. After then, parasites number began to decline. This was also similar to another (18) finding.

Diagnosis of vaginal trichomoniasis based on the clinical examinations had 88% false negative and 29% false positive results (22). This was in accordance with our study finding, although, 37% of pregnant women had vaginal discharge (111/300), the percentage of positive culture was only 17.1% (19/111). This could be explained as clinical symptoms of trichomoniasis may be similar to those of another sexually transmitted disease (23). Moreover, 189 (63%) of pregnant women had normal vaginal findings, 16(8.4%) of them were positive for infection.

Several studies suggested the association between vaginal trichomoniasis and preterm delivery as well as low birth weight infants (3, 24, 25). Pregnant women infected with *T. vaginalis* were 30% more likely than the uninfected women to have preterm delivery or low birth weight infants (3). In addition, the meta-analysis study (26) was confirmed this relationship. That was in accordance with our finding. The high percentage of pre-term delivery and post-delivery adverse outcome observed among the infected women could be explained as the host inflammatory response to infection may reduce chorioamnionitis membrane strength and increase the risk of adverse birth outcomes (27).

All positive cases were treated with Metronidazole; only 6 cases (17.1%) gave birth to normal baby. Metronidazole exposure during pregnancy with trichomoniasis was combined with an increased risk of preterm birth and low birth weight (28, 29). “Metronidazole is an FDA Pregnancy Category B drug: animal reproduction studies have failed to demonstrate a risk to the fetus and there are no adequate and well-controlled studies in pregnant women” (30). In addition, metronidazole has been found to cross the placenta and to induce mutagenicity in bacteria and carcinogenicity in experimental animals (25). Besides, reinfection or metronidazole-resistant strains could be another cause of this adverse pregnancy outcome.

In the present study, the high rate of trichomoniasis was observed in age group of [20–30] yr followed by age group of [31–40]. This finding was consistent with other previous studies (31–33). Trichomoniasis as one of the sexually transmitted infection commonly associated with patients at child-bearing ages since this ages are more sexually active (34).

Our study showed that living in rural area, low socio-economic status and primary educational level were significant risk factors for *T. vaginalis* infection. The similar results were reported in other previous studies (3, 35, 36). This may be due to lifestyle and personal hygienic habit, limited health service and lack of awareness of the health education programmers about sexually transmitted infections arranged by the maternal health office in this area. Contrary, women highly educated and with higher socioeconomic status could adopt appropriate hygienic measures such as using vaginal washing and antiseptics after coitus and/or usage of condom by their husband (37).

## Conclusion

Risk factors associated with *T. vaginalis* infection are maternal age with a range of 20 to 30 yr, low socioeconomic level, living in rural area and primary level of education. Women with any one of these risk factors should be screened for trichomoniasis to reduce the incidence of preterm delivery and low birth weight. The combination of wet mount and culture is still the standard approach for more effective diagnosis, especially in asymptomatic patients.
